# Adaptation for Protein Synthesis Efficiency in a Naturally Occurring Self-Regulating Operon

**DOI:** 10.1371/journal.pone.0049678

**Published:** 2012-11-20

**Authors:** Dorota Herman, Christopher M. Thomas, Dov J. Stekel

**Affiliations:** 1 School of Biosciences, University of Birmingham, Birmingham, United Kingdom; 2 School of Biosciences, University of Nottingham, Sutton Bonington, Leicestershire, United Kingdom; 3 Department of Plant Systems Biology, VIB – Ghent University, Ghent, Belgium; Center for Genomic Regulation, Spain

## Abstract

The *korAB* operon in RK2 plasmids is a beautiful natural example of a negatively and cooperatively self-regulating operon. It has been particularly well characterized both experimentally and with mathematical models. We have carried out a detailed investigation of the role of the regulatory mechanism using a biologically grounded mechanistic multi-scale stochastic model that includes plasmid gene regulation and replication in the context of host growth and cell division. We use the model to compare four hypotheses for the action of the regulatory mechanism: increased robustness to extrinsic factors, decreased protein fluctuations, faster response-time of the operon and reduced host burden through improved efficiency of protein production. We find that the strongest impact of all elements of the regulatory architecture is on improving the efficiency of protein synthesis by reduction in the number of mRNA molecules needed to be produced, leading to a greater than ten-fold reduction in host energy required to express these plasmid proteins. A smaller but still significant role is seen for speeding response times, but this is not materially improved by the cooperativity. The self-regulating mechanisms have the least impact on protein fluctuations and robustness. While reduction of host burden is evident in a plasmid context, negative self-regulation is a widely seen motif for chromosomal genes. We propose that an important evolutionary driver for negatively self-regulated genes is to improve the efficiency of protein synthesis.

## Introduction

Negative self-regulation of transcription is commonly seen for transcription factors in many species and has been identified as a ‘network motif’ [1]. The implication is that evolution has repeatedly selected for negative self-regulation, and therefore that this motif is optimizing some form of phenotypic response. Several hypotheses have been posited about what precisely is being optimized. These include: reduction in the random fluctuations (noise) in the abundance of the regulated proteins [2]–[3], or, more subtly, a change in the noise profile of the regulated proteins [4]; speeding up the response time of the production of the regulated proteins [5]; and reduction in the cost to the organism of producing the regulated proteins [6]. Others have shown that negative self-regulation can improve the trade-offs between these objectives, for example noise reduction and speed [7]. These hypotheses have generally been explored either with generic theoretical models [2] [8] or with synthetic systems [9], often using either parameter values or experimental conditions that do not reflect the *in vivo* operational context of these systems.

We consider a naturally occurring, negatively and cooperatively self-regulated transcription circuit, the central control operon (CCO) of RK2 plasmids. IncP-1 plasmids are broad host range plasmids [10] that have been found in both clinical [11] and environmental bacteria [12]. They frequently carry genes for antibiotic resistance or catabolic pathways [13]. The archetypal IncP-1α plasmid RK2 is a well-characterized biological system, with a fully sequenced and annotated genome [14] and wide range of experimental measurements [15]. Its central control operon encodes the two global regulators KorA and KorB, that cooperatively regulate the operon as well as all of the processes necessary for the plasmid life cycle, including conjugation, replication and partitioning [16].

Within a plasmid context, the possible evolutionary adaptations for negative self-regulation can be articulated as follows. First, the RK2 plasmid is able to persist in most Gram-negative bacteria [10]; these hosts provide different environmental conditions for the plasmid, including availability and specificity of RNA polymerase and ribosomes, and it is plausible that the plasmid has evolved to be robust to changes in extrinsic (host) factors. The second hypothesis is evolution to minimize protein fluctuations: high levels of fluctuations could give an unpredictable response, drawing more host resources when protein levels are high, or reducing plasmid efficacy when protein levels are low. The third hypothesis is that the network has evolved to ensure that, post-conjugation to a new host, the plasmid can establish its transcriptional programme as rapidly as possible. This will ensure replication and transfer capabilities are in place prior to host cell division or even establishment of competitor plasmids [17]. Finally, plasmids need to evolve minimal cost to their host. Thus the fourth hypothesis is that the gene regulatory network has evolved to ensure that the synthesis of plasmid proteins is as efficient as possible in its use of host resources.

More generally, however, cooperative and negative self-regulation is widely observed on chromosomal genes outside of a plasmid context. Thus it is relevant to consider this particular system as a well-characterized example of the network motif, and exploration of these hypotheses in this system will have general application to the understanding of negative self-regulation of other genes.

In previous work, we have developed a mathematical model that we have used to integrate diverse experimental measurements on the RK2 CCO [18]. That model includes the dynamics of protein synthesis, including transcription regulation, protein synthesis, protein dimerization and protein dilution due to cell growth. By expressing the mechanisms as a series of ordinary differential equations, and employing appropriate parameter inference techniques, we were able to estimate all mechanistic parameters in the model, and use them to integrate experimental measurements of protein abundance [19]–[20] and regulatory strength [21].

In this study, we build on our previous experimental and modeling work to investigate the adaptive benefits of the regulatory circuit. We extend the published model to include host growth and replication, plasmid division, molecular noise associated with plasmid gene regulation, and, where appropriate, the synthesis of mRNA. The next step is to build four versions of the model with decreasing complexity. The first model is for the wild-type CCO. The second model is slightly simpler, retaining both regulators, but removing the cooperativity between KorA and KorB; this has been achieved experimentally, by mutating a single amino acid residue, Tyrosine 88 on KorA [22]. The third model is simpler still, considering negative self-regulation by only a single (dimeric) regulator, rather than two regulators. The fourth model considers the simplest system, in which there is no regulation at all, so that all gene expression is constitutive.

We compare each of these four models in the context of each of the four hypotheses posited for the adaptive value of negative self-regulation. Where the addition of regulatory complexity leads to improvements in a measure associated with one of the hypotheses, then that hypothesis is considered to be a good explanation for that regulatory complexity. Conversely, where there is little improvement, then that hypothesis has less explanatory value.

The advantage of this approach is two-fold. On the one hand, our model is grounded in a natural biological system, with extensive experimental measurements and with realistic parameters that have been validated against the experimental data [18]. Thus the results obtained should compare favourably with those associated with purely theoretical models without the same level of biological underpinning, or models associated with synthetic operons. On the other hand, this stochastic and multi-scale model allows us to carry out *in silico* investigations of the four hypotheses posed above that, some of which would be extremely difficult to carry out experimentally. This is particularly the case for mRNA production, since the plasmid transcripts are rare and short-lived, and therefore difficult to observe.

### Model Description

**Figure 1:**
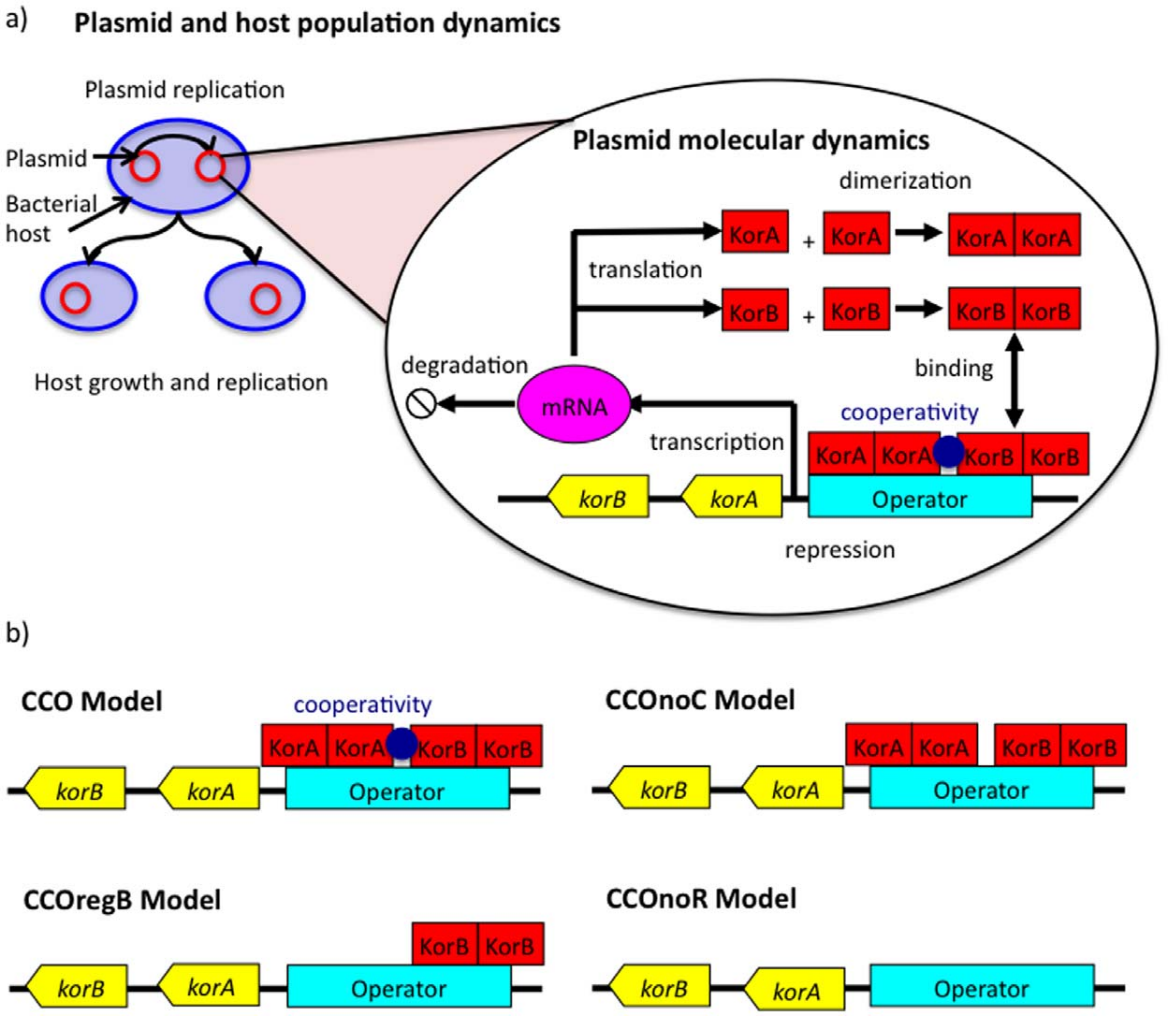
Model representation. (a) Multi-scale stochastic model of gene regulation of the RK2 central control operon. The population dynamic layer contains plasmid replication as an independent Poisson process and continuous host cell growth at an exponential rate with cell division when the cell doubles its initial size. After each division, only one daughter cell is tracked. The plasmid molecular dynamics layer contains protein synthesis, dimerization, cooperative binding of KorA and KorBdimers to the promoter, and repression of the operon. (b) The four models for comparison: CCO is the wild type system; CCOnoC is a system with two regulators but with no cooperativity between then regulators; CCOregB is a system with just a single dimeric regulator; CCOnoR is a system with no regulation. In order to ensure comparability, the rates of protein synthesis are tuned so that protein abundance is the same in all models (see *Methods*). (c) Stochastic model formulation for the CCO model: A_1_, A_2_, B_1_, B_2_– KorA and KorB monomer and dimer, respectively; D, X, Y, Z – states of the DNA strand: empty DNA, KorA-DNA, KorB-DNA, KorA-KorB-DNA complexes respectively; k_p_ – plasmid replication rate, k_A_, k_B_ – maximal KorA and KorB synthesis rates, π_X_, π_Y_ - scaling parameters for the protein synthesys, k_onP_ – protein dimerization rate, k_onD_ – protein association rate to the DNA; k_off1_, k_off2_, k_off3_, k_off4_– KorA, KorB dissociation rates, KorA for KorA-DNA complex, KorB from KorB-DNA complex, KorA from KorA-KorB-DNA complex and KorB from KorA-KorB-DNA complex, respectively. Each of the other models is derived by appropriate simplification of this model. For CCOnoC, k_off1_ = k_off3_ and k_off2_ = k_off4_; for CCOregB, the reactions between KorAdimers and DNA are removed from the model; for CCOnoR, the reactions between KorBdimers and DNA are also removed from the model.

The system is modelled using a multi-scale approach that includes plasmid and host replication as well as the dynamics of plasmid gene regulation (Figure 1); the approach includes both deterministic and stochastic components. Details of parameter values and simulation techniques are provided in the Methods section.

In all versions of the model, plasmid replication is considered as an independent Poisson process with a fixed rate k_p_. In reality, this behaviour derives from tight molecular control of plasmid replication, but such control is beyond the scope of this model, so we make use of the emergent phenotype. Host cell growth, also in all versions of the model, is deterministic and exponential according to the equation:
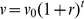
(1)


Each time the host cell volume reaches the value of 2v_0_ the host cell divides; thus cell divisions occur at fixed time intervals every ln(2)/r seconds. After cell division, the cell contents are divided between the daughter cells.

### Main Model

The main model contains the full regulatory mechanism of the CCO, based closely on our previous work [18], and is used for the comparison of robustness, protein fluctuations and response times. The other model variants are derived from this model, and are distinguished by their consideration of slightly different sets of molecular reactions. In the main model, the KorA and KorB proteins form homodimers, and when both are bound to the operator, the operon is fully repressed. The operon is fully expressed when neither transcription factor is bound to the operator. Partial repression occurs when either KorA or KorB dimers bind in the absence of the alternate regulator. Cooperativity is achieved by having separate parameters for KorA and KorB dimers dissociating from the DNA, according to whether the alternate regulator is also bound. The stochastic (chemical) equations are:
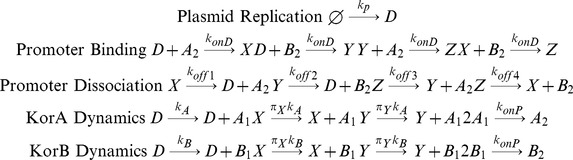
(2)


In Equations 2, A_1_, A_2_, B_1_ and B_2_ represent the KorA and KorB monomer and dimer, respectively; D, X, Y and Z represent states of the DNA strand: empty DNA, KorA-DNA, KorB-DNA and KorA-KorB-DNA complexes respectively. k_p_ is the plasmid replication rate, k_A_ are k_B_ the maximal KorA and KorB synthesis rates, π_X_ and π_Y_ are scaling parameters for the protein synthesis associated with partial repression, k_onP_ is the protein dimerization rate, k_onD_ is the protein association rate to the DNA, k_off1_, k_off2_, k_off3_ and k_off4_ are the KorA, KorB dissociation rates: KorA from KorA-DNA complex, KorB from KorB-DNA complex, KorA from KorA-KorB-DNA complex and KorB from KorA-KorB-DNA complex respectively. The on-rate parameters k_onP_ and k_onD_ are inversely proportional to host volume. This dependence reflects changes in diffusion time as a consequence of larger cell volume. All other parameters are independent of host cell volume.

### Comparator Models

The three simpler versions of the model differ in terms of regulation of the CCO as a progression of models that decrease in complexity. For the non-cooperative model, denoted CCOnoC, the affinities of the KorA and KorB dimers are unchanged by the bound alternate regulator: the equations are the same as in Equations 2, but the parameter k_off3_ is set to be equal to k_off1_, and the parameter k_off4_ is set to be equal to k_off2_.

For the model with a single regulator, denoted CCOregB, the reactions between KorA dimers and the operator are removed from the model. Thus the chemical equations are:
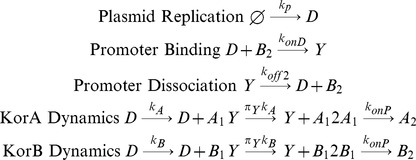
(3)


For the model without regulation, denoted CCOnoR, the reactions both between KorA dimers and the operator and between KorB dimers and the operator are all removed from the model. Thus the chemical equations are:
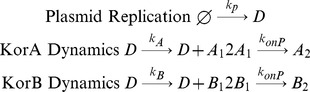
(4)


In all three cases, the host cell growth, host cell division and plasmid division elements of the model are unchanged. In the case of analyses of protein fluctuations, additional simulations were carried out for models in which the plasmid copy number was held constant, by removing the plasmid replication event from the main model and all three comparator models.

### Model that Includes mRNA Synthesis

For the comparison of mRNA synthesis between the four schemes, a slightly more complex set of models is used that includes separate mRNA and protein synthesis steps. This model is necessary for the exploration of this hypothesis, but has the disadvantage that the parameters introduced for the separate steps of transcription, translation and mRNA turn-over have not been separately estimated from the experimental data. The equations are:
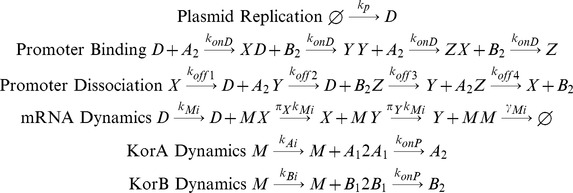
(5)


These equations contain mRNA as a new species. Synthesis of mRNA is modelled in an analogous fashion to the protein synthesis steps in the main model, at maximal rate k_Mi_. mRNA degrades at rate g_M_; this is included into the model as mRNA degradation is fast relative to cell growth. The mRNA M encodes both the *korA* and *korB* genes and so translation of each protein can occur from the mRNA, at rates k_Ai_ and k_Bi_ respectively. For the comparison of mRNA synthesis, comparator models have been derived from this model in an analogous fashion to the comparator models derived above.

### Evolutionary Trajectory Analysis Model

For the trajectory analysis, we consider a model with a single transcription factor that operates as a partial repressor, analogous to KorA or KorB, while including mRNA dynamics. We explore behaviour of this model as two parameters are varied: the partial repression parameter, that controls the extent to which the transcription factor blocks gene expression; and the affinity of the transcription factor to the DNA. The equations are given by:
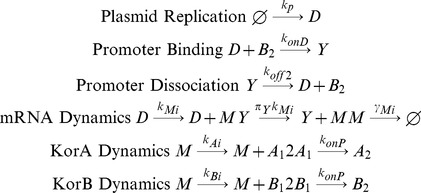
(6)


### Model Comparisons

For comparing the four models, we have tuned the protein synthesis rates for each model in order to ensure equal values of protein abundance (Table S2). In doing so, we assume that the set level of KorA and KorB has evolved to concentrations associated with their global regulatory functions in all aspects of the plasmid life cycle. For three cases, this is controlled by a single parameter, the protein synthesis rate. However, for the study of mRNA production analyses, we have considered two scenarios. The first is to tune the translation rate whilst holding the transcription rate constant; in this case, the models with simpler regulatory mechanisms also have lower maximal translation rate (Table S3). The second scenario is to tune the transcription rate, whilst holding the translation rate constant. In this case, the models with simpler regulatory mechanisms also have lower maximal transcription rates (Table S3). Evolution might use a combination of both sets of changes; these two scenarios represent the two extreme possibilities and thus are useful for model comparison.

For the evolutionary trajectory analysis, two parameters are varied. In order to ensure equal values of protein abundance, the translation rate is tuned (Table S4) while the transcription rate is held constant.

## Results

### Increased Regulatory Complexity Provides Limited Improvement in Robustness to Factors Impacting on Protein Synthesis

Sensitivity analysis of the four architectures to changes in each of the parameters, as measured by concentration control coefficients of the protein concentrations, are summarized in Table 1. A decrease in control coefficients between the models represents an increase in robustness for that model. For most of the parameters that are present in all models, there is minimal change in robustness as the regulatory complexity increases. Note the synthesis rates k_A_ and k_B_ are parameters that represent an amalgamation of all processes involved in protein synthesis, including transcription, translation and mRNA turn-over, so the different systems are similarly robust to changes in rates of any of these processes. The one exception is the rate of plasmid replication, k_p_, where the concentration control coefficient of KorA in the systems regulated by two repressors with or without cooperativity (0.21, 0.22) is half that of systems with a single regulator or the unregulated system (0.50, 0.54). However, there is little impact of cooperativity relative to the non-cooperative model.

**Table 1:** Concentration control coefficient of KorAdimers for each parameter of the models.

	CCO	CCOnoC	CCOregB	CCOnoR
k_off1_	0.005	−0.001	–	–
k_off2_	−0.004	0.004	0.040	–
k_off3_	0.056	0.058	–	–
k_off4_	0.160	0.146	–	–
k_A_	0.431	0.431	0.560	0.471
k_B_	−0.112	−0.104	0.007	−0.014
k_P_	0.208	0.215	0.544	0.494
π_X_	0.174	0.160	–	–
π_Y_	0.050	0.060	0.537	–
k_onD_	−0.151	−0.144	0.025	–
k_onP_	−0.002	0.002	0.040	0.001

k_off1_, k_off2_, k_off3_, k_off4_– KorA, KorB dissociation rates, KorA for KorA-DNA complex, KorB from KorB-DNA complex, KorA from KorA-KorB-DNA complex and KorB from KorA-KorB-DNA complex, respectively, k_A_, k_B_ – maximal KorA and KorB synthesis rates, k_p_ – plasmid replication rate, π_X_, π_Y_- scaling parameters for the protein synthesis, k_onD_ – protein association rate to the DNA, k_onP_ – protein dimerization rate. Smaller control coefficient implies greater robustness; model descriptions in Figure 1b. Note that the models have similar robustness for the majority of parameters; this includes the parameters governing protein synthesis and protein-DNA affinity and cooperativity. The one exception is the plasmid replication rate; the systems with two regulators are more robust than the systems with zero or one regulator.

### Negative and Cooperative Self-regulation has Limited Impact on Protein Concentration Fluctuations

**Figure 2:**
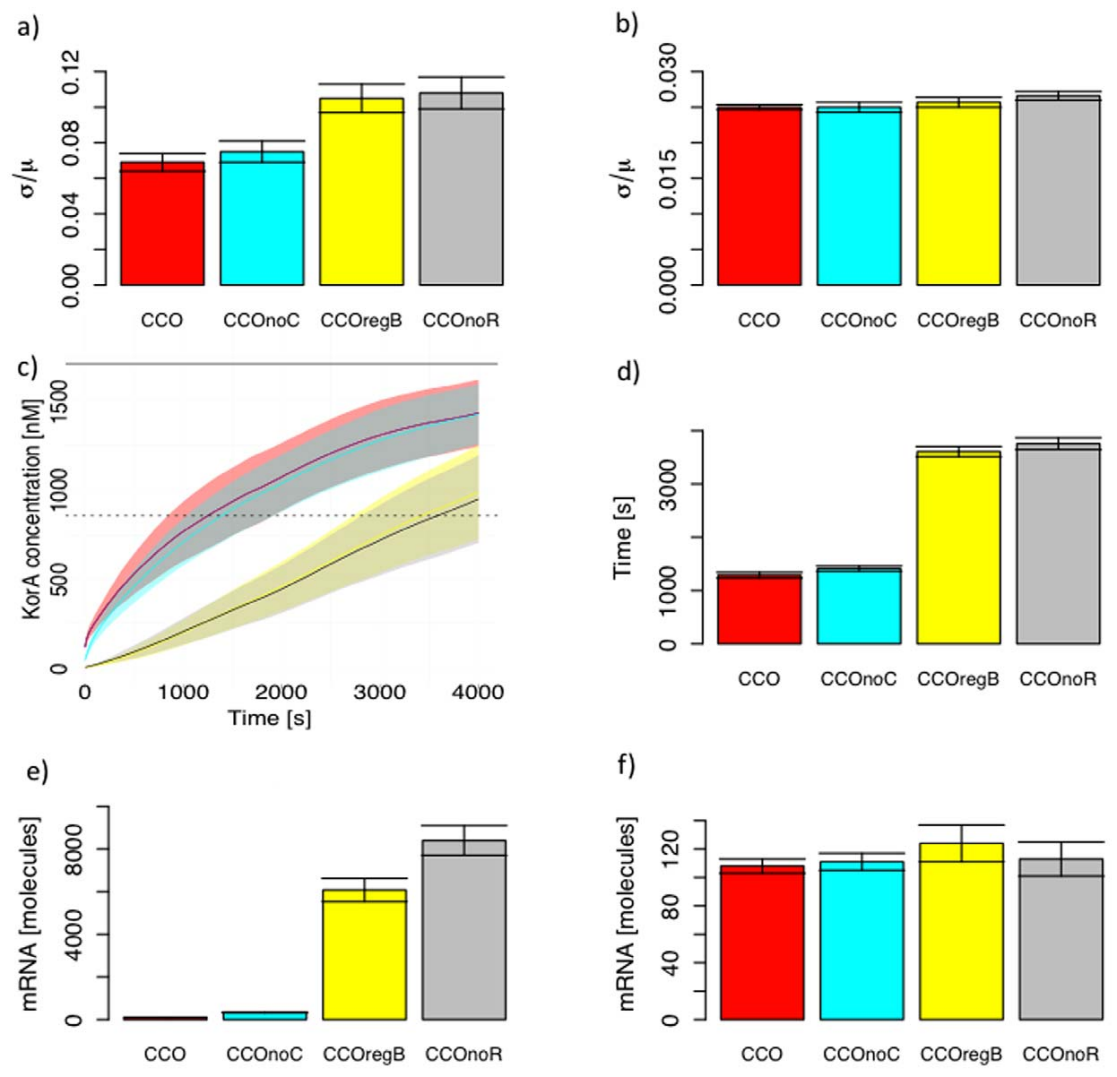
Results on the various optimizations by the central control operon regulation. Comparison of the ability of the four models to optimize different desirable properties. With the exception of (c), bar heights are means and error bars are standard errors across 20 replicates. (a) Fluctuations in KorB regulator concentration in their steady state for models that include plasmid replication and (b) exclude plasmid replication. There is little improvement in protein fluctuations between the models, with a minor improvement observed when a second regulator is introduced when plasmid copy number fluctuations are also present. (c) Dynamics of KorA total monomer concentrations after a new host transfection; means of arising concentrations over time are indicated by solid lines, the shadows show standard deviations. The horizontal solid line indicates a mean KorA concentration and dashed line a half of the mean concentration. (d) Times of reaching a half of a mean KorA concentration with standard errors; the systems with strong regulation reach a half of mean KorA concentration quicker than with weak or without regulation. (e) Number of mRNA produced per generation after the model has reached steady state. CCOnoR (grey) has no regulation; CCOregB (yellow) has a single regulatory protein; CCOnoC (cyan) has two regulatory proteins; CCO (red) has two regulatory proteins with cooperativity. The translation rate is tuned so that the total protein abundance is held constant. There is a small reduction in mRNA usage after the introduction of a single regulator; a second regulator brings a 20-fold improvement in mRNA usage; the introduction of cooperativity brings a further 3-fold improvement. (f) The transcription rate is tuned so that protein abundance is held constant. All four architectures are equivalent.

There is a small decrease in fluctuations in protein abundance from the mean steady state level, as measured by the coefficient of variation, between models with no regulator or a single regulator (c.v. = 0.11) and models with two regulators (c.v. = 0.07) (Figure 2a). There is no significant difference between the models with and without cooperativity. Figure 2b shows the fluctuations of protein abundance in the models where plasmid copy number is held constant: there are no differences in fluctuations between the four models (c.v. approx. 0.025). Thus it appears that the decrease in fluctuations of protein abundance seen between the models is due to the interaction between gene regulation and fluctuations in plasmid copy number. Overall, self-regulation has limited impact on the magnitude of protein fluctuations and cooperativity has no impact.

### Strong Negative Auto-regulation Speeds up the Accumulation of Regulatory Proteins

Figure 2c shows the increase in KorA concentration from plasmid RK2 following transfection to a new host; Figure 2d shows the time taken to reach half of the mean KorA concentrations at steady state. The models with the slowest dynamics are models with either no regulator or a single regulator, taking 3754 or 3606 seconds respectively. The most competitive models are models with two regulators: the model without cooperative regulation takes 1412 seconds and the model with cooperative regulation takes 1288 seconds. Thus the naturally occurring system shows greater than two-fold improvement in response time relative to the unregulated system or a system with a single regulator. However, there is only a minimal improvement in response time in systems with our without cooperativity, although this improvement is statistically significant (p<<0.001).

### Cooperative and Negative Auto-regulation Substantially Improves the Efficiency of Protein Synthesis

There is a considerable reduction in the number of mRNA molecules produced per cell cycle between the four model systems, in the scenario where the translation initiation rate is tuned (Figure 2e). A similar result is seen when considering the first ten generations post-conjugation (Figure S1). The unregulated system produces an average of 8402 mRNA molecules per host generation. By introducing a single regulator, production reduces to 6077 molecules per generation. The introduction of a second regulator reduces the number of mRNA molecules more than 20-fold to 331 molecules per generation. The introduction of cooperativity between the two regulators, so that the system has the complexity of the wild-type plasmid, results in a further 3-fold reduction to 107 mRNA molecules per generation. On the other hand, when controlling protein abundance by tuning transcription initiation, there is no impact on the numbers of mRNA molecules produced per cell cycle (Figure 2f).

The relative costs of transcription and translation in each of the four models can be compared directly by considering the number of phosphate groups (e.g. from ATP) required per cell cycle. The KorA protein is 101 amino acids and the KorB protein is 359 amino acids. Protein synthesis costs approximately 4 phosphates (∼P) per a.a. [23]. There are 4000 KorA molecules and 1000 KorB molecules per cell [19]–[20]; in a single cell cycle, half of these would need to be replaced, so the replacement cost per cell cycle is approximately 1.53×10^6^ ∼P. This cost is assumed to be the same in all four models. Considering transcription, the total length of transcript associated with KorA and KorB (not including the other genes on the operon) is 1380 nucleotides, and the cost is 2 ∼P/nucleotide [23]. Thus in the four models considered, the total cost of mRNA production for KorA and KorB per cell cycle are 2.3×10^7^ ∼P for no regulation, 1.7×10^7^ ∼P with a single regulator, 9.1×10^5^ ∼P with two regulators, and 3.0×10^5^ ∼P including cooperativity. Overall, the CCO regulatory mechanism produces the same set level of protein for less than 10 times the total cost as compared with the unregulated system (Table 2).

**Table 2:** Energetic analysis of cost of transcription and translation for each of the models.

Model	mRNA molecules/cell cycle	Transcription Cost/∼P	Translation Cost/∼P	Total Cost/∼P
CCO	107	2.95×10^5^	1.53×10^6^	1.82×10^6^
CCOnoC	331	9.14×10^5^	1.53×10^6^	2.44×10^6^
CCOregB	6077	1.68×10^7^	1.53×10^6^	1.83×10^7^
CCOnoR	8402	2.32×10^7^	1.53×10^6^	2.47×10^7^

The cost of protein synthesis for each model taking account the number of mRNA molecules produced during one cell cycle and translation to replace half of the total KorA and KorB protein pool during a cell cycle. The cost is calculated in terms of phosphate groups from ATP. In the unregulated model, he transcription cost is more than 5-fold greater than the translation cost. This reduces to less than 7% of the translation cost in the fully cooperative model. Thus the overall benefit of the cooperative regulation is a greater than 10-fold reduction in cost to the host of producing the same quantity of protein.

### Results Presented are Robust to Uncertainty in Parameter Values

**Figure 3:**
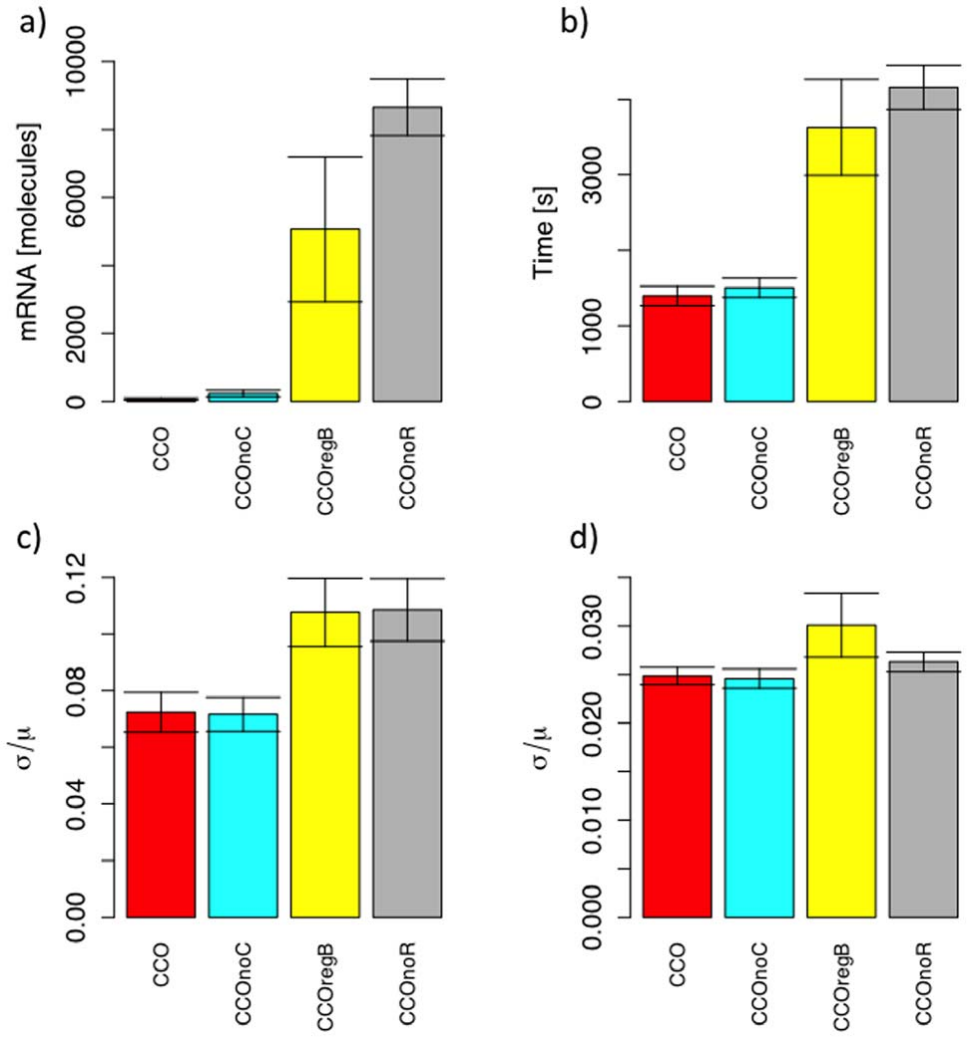
Robustness of central control operon regulation results. Comparisons between the different models are robust to uncertainty in parameter values. Bar heights are means; error bars are standard deviations from 1000 resamples of the parameter values. (a) Differences between the models in control of protein fluctuations show a similar pattern to Figure 2(a), demonstrating that the result is robust to uncertainty in parameter values. (b) A similar pattern is plasmid replication is included, as per Figure 2(b). (c) Difference in response times between the four models is comparable to that shown in Figure 2(d). Note, however, that the difference in mean response time between the models with and without cooperativity is small relative the variability due to uncertainty in parameter values. (d) Difference in mRNA usage between the four models when transcription rate is held constant shows the same overall result as Figure 2(e). The difference between the CCOnoC and CCO model results are more than 3-fold, comparable with Figure 2(e), and which is difficult to see in the bar plot. A comparison with translation rate held constant, as per Figure 2(f), is shown in Figure S2.

The results presented above are based on a single representative parameter set that optimally fits the data, as derived from our previous work [18]. However, in that work, we also derived posterior distributions for each of the parameter values that represent our uncertainty in those values. In order to test whether our results are robust across a range of realistic possible parameter values, we re-sampled from those posterior distributions (described in *Methods*) and ran the comparisons for 1000 different sets of parameter values. The model comparisons for protein fluctuations, response times and mRNA usage (transcription constant) are shown in Figure 3; the results are broadly similar to those described above, demonstrating that these are robust to uncertainty in parameter values. The only minor difference is in the response time comparison between the models with and without cooperativity: although the mean response time with cooperativity is still lower, the improvement is small relative to the variability due to uncertainty in parameter values. The model comparisons in mRNA usage with translation constant also give similar results (Figure S2). Similarly, the results on robustness to changes in the parameter values are themselves robust to variability in the underlying parameters: the mean control coefficients are almost identical to those presented in Table 1 and are presented in Table S1.

### A Simplified Model Suggests an Evolutionary Trajectory from an Unregulated System to the Wild-type Architecture

In order to ascertain how the wild-type system may have evolved, we consider a simplified version of the model with just a single regulator. Negative repression can be strengthened either by increasing the level of repression when the repressor is bound, or by the increasing the strength of the repressor-DNA binding. The first of these is analogous to the wild type mechanism of having two different repressors acting in different ways to repress transcription (KorA competing with RNA polymerase and KorB preventing opening of the DNA); the second of these is analogous to the evolution of cooperativity between the two transcription factors.

**Figure 4:**
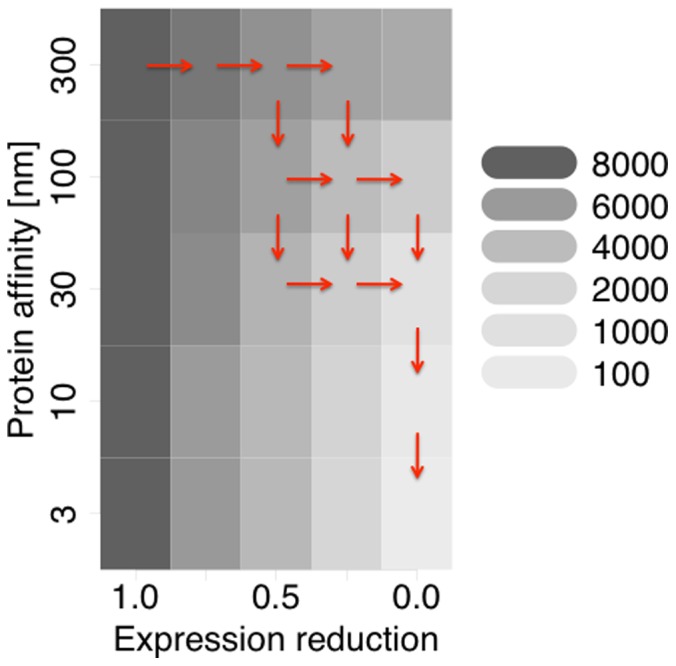
Optimization of the mRNA production by the central control operon regulation. mRNA production per one generation for a single negative regulation for different repressor-DNA affinities and different levels of reduction in expression while a repressor is bound to the DNA. The red arrows represent statistically significant differences of mRNA production. The top-left hand corner approximates an unregulated system while the bottom-right corner approximates the wild-type system of RK2. The implied evolutionary trajectory is that first increased efficacy of repression would evolve following which the protein-DNA interaction would become stronger.

We measured the number of plasmid mRNA molecules produced by the host per host generation, as a measure of cost to host, for a range of expression reduction and protein-DNA affinities (Figure 4). The red arrows show a possible evolutionary path indicated by the greatest decrease in mRNA production from one state to another. More than one arrow from one state to another indicates lack of statistical difference between numbers of mRNA produced for competing states. The path indicates that evolution would first favour a reduction in expression from the regulated DNA, followed by an increase in regulatory binding strength. This would suggest that the wild type architecture would first have evolved to increase repression by blocking both RNA polymerase and DNA melting, following which selection would have favoured cooperativity as a means to strengthen the protein-DNA interaction.

## Discussion

In this work, we have carried out a detailed analysis of the architecture of a naturally occurring negatively and cooperatively self-regulated operon, the *korAB* operon in RK2 plasmids. We have used a multi-scale stochastic model to unravel how the different elements of the regulation impact on possible evolutionary advantages of the gene regulatory architecture. Specifically, we have investigated four hypotheses associated with the function of negative self-regulation: improving robustness, minimizing protein fluctuations, minimizing response times and improving efficiency of protein synthesis. We have shown that, at least with this system, the most significant improvements are seen in improving efficiency of protein production. Improvements are seen in response time, but only for stronger regulation and not for cooperativity. The gene regulatory architecture conveys only a small advantage in reduction of protein fluctuations or robustness to extrinsic factors, both in relation to the plasmid replication rate.

These results can be viewed in the specific context of the biology of RK2 plasmids; but they also extend to the study of the evolutionary role of negative and cooperative self-regulation. Negative self-regulation has been identified as an extremely common network motif for chromosomal genes in both prokaryotic and eukaryotic cells. From a plasmid perspective, it is expected that there would be strong selection for minimization of host burden, so increased efficiency of protein synthesis would contribute to this goal. Indeed, the RK2 plasmid has evolved to persist in a wide range of hosts, and it is possible that its elaborate mechanisms of gene regulation have evolved to enable it to be a low burden in a wide range of host contexts. However, efficient protein production is just as important for a host cell as it is for a plasmid [24], and in this work we posit that an important evolutionary adaptation of negative self-regulation is to minimize the metabolic cost of protein production. In previous work, we have shown that artificial regulatory networks that are free to evolve either their topology or their parameters have evolved negative self-regulation for this same reason [25]. The results of this study on a natural system strongly support this position.

The reduction in mRNA usage is achieved by allowing translation in the cooperative model to be more efficient than it is in models with less regulatory complexity. It could be argued that increased translational efficiency is an additional cost not factored into the model, for example by drawing ribosomes that could otherwise be used elsewhere in the host. However, because we have controlled the protein abundance to be the same in all models, the mean ribosome usage per cell cycle is identical in all the systems: what differs is that in systems with more complex regulation, the ribosomes are required in larger numbers for a short period of time, while in systems with less or no regulation, the requirement is for more dispersed availability over the cell cycle. This irregular, bursty requirement for ribosomes in the wild type system is likely to be more consistent with host use of ribosomes; it is known that transcription is bursty [26], and with many host proteins also being negatively self-regulated, these genes too would also have requirements for larger numbers of ribosomes for short periods of times. Thus these results are consistent with a picture of protein synthesis in which different transcripts are being manufactured at different times, and ribosomes are being recruited in numbers to different operons as they are required.

The results on mRNA usage can also be explained with the help of a toy ODE model. In its simplest form, models for gene regulation could be expressed by two equations:
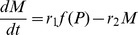
(7)

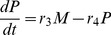
(8)


The function *f(P)* satisfies 0<*f(P)* ≤1 and encapsulates the mechanisms of gene regulation, including possible protein dimerization and transcription regulation. In the special case of no regulation, *f(P)* = 1 and the solution is:
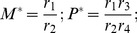
(9)


Now consider a system with some transcription regulation so that *f(P)*<1. In order to make the comparisons, we need to tune parameters so that the protein abundance is still the value P^*^. This could be achieved by tuning the transcription rate r_1_ or the translation rate r_2_. If we hold the translation rate fixed and tune the transcription rate r_1_, then we obtain a new value r_1_ that satisfies r_1_f(P*) = *r_1_*. The mRNA abundance satisfies.
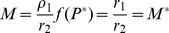
(10)and so is unchanged relative to the unregulated model, no matter how strong the regulation. If, on the other hand, we hold transcription constant, we tune translation rate to obtain a new value *r_3_*, then r_3_f(P*) = *r_3_*. The resulting mRNA level thus satisfies



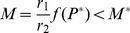
(11)Thus mRNA abundance is decreased relative to the unregulated model. The stronger the mechanism of repression, the smaller the value of f(P^*^), and so the less mRNA is required to produce the same quantity of protein. The same argument holds for mRNA production dM/dt.

**Table 3:** Parameter values.

Parameter	Value	Parameter	Value
k_off1_	1.29 [s^−1^]	k_onP_	0.0003–0.0006 [s^−1^]
k_off2_	0.93 [s^−1^]	k_A_	11.5 [s^−1^]
k_off3_	0.31 [s^−1^]	k_B_	3.2 [s^−1^]
k_off4_	0.31 [s^−1^]	k_p_	0.0045 [s^−1^]
k_onD_	0.03–0.06 [s^−1^]	vol	2.77–5.54 [µm^3^]

k_off1_, k_off2_, k_off3_, k_off4_,- protein dissociation rate, KorA from a KorA-DNA complex, KorB from a KorB-DNA complex, KorA from a KorA-KorB-DNA complex and KorB from a KorA-KorB-DNA complex, respectively, k_onD_ – protein association rate to the DNA strand, k_onP_ – KorA and KorBdimerization rate, k_A_, k_B_ – KorA and KorA synthesis rate for a CCO model, respectively, k_p_ – plasmid replication rate, vol – host cell volume.

**Table 4:** Parameter value uncertainty associated with resampling regime.

Parameter	Prior	Approximate Marginal Posterior Distribution	Mean	Standard Deviation
k_off1_	Informative	Normal	13.16	1.3
k_off2_	Informative	Normal	9.7	0.95
k_off3_	Informative	Normal	3.08	0.3
k_off4_	Informative	Normal	2.97	0.3
k_A_	Uninformative	Lognormal	19.5	4.2
k_B_	Uninformative	Lognormal	5.5	1.16
π_X_	Uninformative	Uniform	0 (minimum)	1 (maximum)
π_Y_	Uninformative	Uniform	0 (minimum)	1 (maximum)
G	Informative	Normal	0.00039	0.00002
λ_A2_	Informative	Normal	0.001	0.00005
λ_B2_	Informative	Normal	0.001	0.00005

Uncertainty in parameter values derived from previous work [18], conditional on KorA and KorB abundance predicted to be within 10% of their required values. For seven of the parameters, inference was carried out with informative prior distributions reflecting experimental observations, and the marginal posterior distributions for these parameters show similar means and smaller standard deviations than the prior distributions. For the protein synthesis rates k_A_ and k_B_, for which uninformative priors were used, the parameter estimates show an uncertainty of approximately 20%. For the scaling parameters π_X_ and π_Y_, the marginal posterior distributions are approximately uniform across the range [0,1].

The relative lack of reduction in protein fluctuations appears to be a surprising result, especially given considerable literature that suggests that negative self regulation acts to decrease protein fluctuations [2]–[3]. However, in previous work, we have also shown that physiologically strong negative regulation can potentially increase protein fluctuations by enhancing “burstiness” - [6]. In the case of this system, it would appear that control of protein fluctuations is not important; it is entirely plausible that in other biological contexts the control of fluctuations may have greater importance.

The lack of impact of gene regulatory network architectures on robustness is a particularly surprising result. In the model used, the protein synthesis parameters (k_A_ and k_B_) amalgamate many processes, including RNA polymerase availability, transcription initiation, translation and mRNA turn-over. Thus these parameters are likely to vary considerably in different hosts, and it is plausible that the gene regulatory network may have evolved in order to ensure that the plasmid can respond effectively in a range of hosts. However, we have shown in Table 1, that protein abundance responds similarly to changes in these parameters in all the models, i.e. the CCO system with strong regulation and a strong promoter is similarly robust to changes in these parameters as the CCOnoR system with no regulation and a weak promoter.

An important consideration of this work is the use of computational models to explore hypotheses that would be difficult or expensive to carry out experimentally. While, for example, response times are straightforward to measure, the production of rare mRNAs is difficult to measure, especially in plasmid systems. In the case of the wild-type RK2 plasmid, we predict that approximately 100 *korAB* mRNA molecules would be produced per host cell generation, so the number of mRNA molecules present would be very low. This work provides a clear prediction: that the abundance of plasmid mRNA produced by hosts bearing wild-type and mutant RK2 plasmids would be very different; this could justify the expense of carrying out experimental work to verify this point.

## Methods

### Parameter Values

The majority of the parameter values have been derived from our previous work [18] in which a deterministic version of the model was carefully analyzed in the context of experimental measurements of the system; for this work, the parameters have been redefined in units of molecules per cell as relevant to stochastic simulations (Table 3). The mean cell volume (4.15 µm^3^) is implicit in the calculations in the experimental work in which KorB total monomer abundance (1000 molecules) and concentration (400 nM) were given [20]. Host cell doubling time of 1789 seconds has been calculated from a host growth rate of 1/43 minutes (unpublished data from Thomas). The exponential function for the growth of the host cell (Equation 1) is based on the mean cell volume and host doubling time. Plasmid replication rate has been tuned in order to obtain a mean plasmid copy number 11–12, which corresponds to 4–7 copies per chromosome (the measured quantity). For protein-protein interactions (dimerization), KorA and KorB are considered as medium large particles with diffusion limited reaction rate of 10^6^ M^−1^s^−1^ [27]. The protein-DNA interaction rate is set 2 orders of magnitude faster as 10^8^ M^−1^s^−1^ [28]. The conversion of these parameters into the stochastic model takes into account the multi-scale nature of the model, as the stochastic reaction rates derived from these parameters are volume dependent and so depend on the timing of the reaction in the host cell cycle.

For comparing the four models, we have tuned the protein synthesis rates for each model in order to ensure equal values of protein abundance (Table S2). For the study of mRNA production analyses, we have considered two possible tunings of the models, tuning the transcription or translation rates (Table S3). For analyses of the simplified model, two parameters are varied. In order to ensure equal values of protein abundance, the translation rate is tuned (Table S4) while the transcription rate (k_Mi_ = 0.4 s^−1^) and mRNA turn-over are held constant (γ_Mi_ = 0.003 s^−1^).

### Simulations and Calculations

The stochastic models were simulated with a hybrid stochastic simulation algorithm. Times for events with constant propensities (i.e. not volume-dependent) are calculated and updated according to a Gibson-Bruck rule [29], with the next event time updated if that event did not occur. Times for events with volume-dependent propensities are calculated according to a Gillespie rule [30]. Timings for fixed cell divisions are handled in a non-Markovian way. For each system we ran 20 simulations, each containing 30 host cell generations. For dynamics analyses we ran 100 simulations for each model. The starting point of each simulation was a single host cell newly transfected with one copy of plasmid RK2, and so without any plasmid products in the system. Cell volume increases exponentially (Equation 1). Cell division occurs each time the cell doubles in size; the cell volume and the quantities of each reactant in the cell are divided by two. When there are an odd number of molecules to divide, say 2k+1 molecules, then each daughter cell inherits either k or k+1 molecules with equal probability. Throughout the whole simulation, we only keep track of one host cell, so after each cell division, one of the two daughter cells is chosen at random and the other discarded.

For the dynamics analyses, we recorded the times at which a half of the mean protein concentration of a particular simulation occurred. For each model, a mean of these times over 20 simulations was calculated. The reported p-value is based on a 2-sample t-test using 100 simulations of each model.

For the fluctuation analyses we considered fluctuations in the concentration of the regulatory protein KorB. KorB was chosen as it is present in lower abundance (∼1000 copies per cell) than KorA (∼4000 copies per cell), so the noise effects, variability relative to mean, are greater. The calculations consider the long term steady state distribution of the protein concentration, between 20^th^ and 30^th^ of host cell generations for each simulation and a mean was taken over 20 simulations.

Robustness analyses were conducted with Stochastic Control Analyses [31] by calculations of stochastic sensitivities for means. The mean values were obtained from over 20 simulations for a model with each parameter changed by 2-fold.

The analysis of the simplified model considers the numbers of mRNA produced per one generation once protein concentration has reached its equilibrium distribution. The mean numbers of manufactured mRNA were calculated over 20 independent simulations for each system, in which the repressor affinity to the DNA strand and expression reduction parameter have been varied (Table S4).

### Robustness to Parameter Value Uncertainty

Our earlier work [18] provided joint posterior distributions for the parameter values used in the model. To evaluate the robustness of results to parameter value uncertainty, we re-sampled from this the posterior distribution conditioned on those values that gave predictions of KorA and KorB protein abundance to be within 10% of their desired values; this condition is necessary to enable fair model comparison. The ranges of parameter values tested are summarized in Table 4. 1000 re-samples from this (joint) distribution were evaluated. All model simulations were carried out as described in previous sections.

## Supporting Information

Figure S1 **mRNA production over 10 first generations.** a) transcription rate is constant, b) translation rate in constant; model descriptions in figure 1b.
(TIF)

Figure S2 **Robustness of mRNA production to parameter uncertainty.** Similar numbers of mRNA molecules generated per cell cycle in each of the four models when transcription rate is tuned and translation rate held constant.
(TIF)

Table S1 **Mean concentration control coefficient of KorA dimers for each parameter of the models.** k_off1_, k_off2_, k_off3_, k_off4_– KorA, KorB dissociation rates, KorA for KorA-DNA complex, KorB from KorB-DNA complex, KorA from KorA-KorB-DNA complex and KorB from KorA-KorB-DNA complex, respectively, k_A_, k_B_ – maximal KorA and KorB synthesis rates, k_P_ – plasmid replication rate, π_X_, π_Y_- scaling parameters for the protein synthesys, k_onD_ – protein association rate to the DNA, k_onP_ – protein dimerization rate. Smaller control coefficient implies greater robustness; model descriptions in figure 1b. These are mean control coefficients from 1000 re-samples of parameter values. Note that the values are very similar to those presented in Table 1, indicating that the results on robustness of the systems to changes in parameter values are themselves robust to uncertainty in the parameter values.
(DOCX)

Table S2 **KorA and KorB synthesis rates for different models.** k_A_ – KorA synthesis rate, k_B_ - KorB synthesis rate; model descriptions in figure 1b.
(DOCX)

Table S3 **Parameter values for mRNA production analyses.** k_Ai_, k_Bi_ – KorA and KorB translation initiation rates respectively, k_Mi_ – transcription initiation rate, γ_Mi_– mRNA turn-over rate; model descriptions in figure 1b.
(DOCX)

Table S4 **Parameter values for analyses of regulatory mechanism evolution.** k_aff_ – an affinity of a transcription factor to the DNA strand, r – expression reduction, k_Ai_, k_Bi_ – KorA and KorB synthesis rates respectively; model descriptions in Figure 1b.
(DOCX)
