# Supplementary figures and images for: Adaptation for Protein Synthesis Efficiency in a Naturally Occurring Self-Regulating Operon
Source: PLoS One. 2012 Nov 20;7(11):e49678. doi: 10.1371/journal.pone.0049678 (PMC3502259; doi:10.1371/journal.pone.0049678)

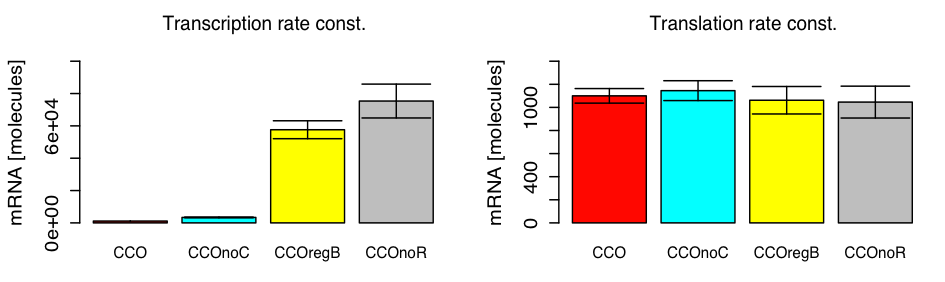

Supplement: Figure S1 — mRNA production over 10 first generations. a) transcription rate is constant, b) translation rate in constant; model descriptions in figure 1b. (TIF) [file pone.0049678.s001.tif]

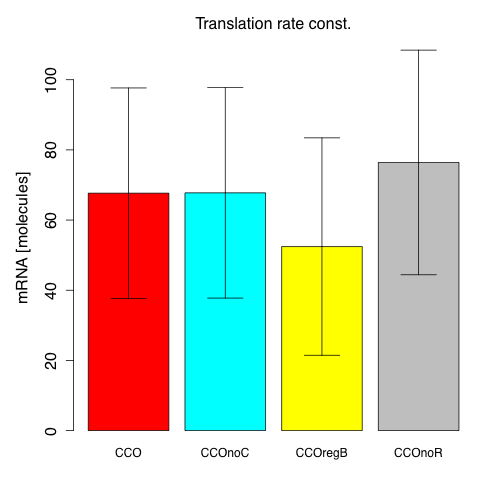

Supplement: Figure S2 — Robustness of mRNA production to parameter uncertainty. Similar numbers of mRNA molecules generated per cell cycle in each of the four models when transcription rate is tuned and translation rate held constant. (TIF) [file pone.0049678.s002.tif]
