# Supplementary material for: Adaptation for Protein Synthesis Efficiency in a Naturally Occurring Self-Regulating Operon
Source: PLoS One. 2012 Nov 20;7(11):e49678. doi: 10.1371/journal.pone.0049678 (PMC3502259; doi:10.1371/journal.pone.0049678)
Supplement: Table S1 — Mean concentration control coefficient of KorA dimers for each parameter of the models. koff1, koff2, koff3, koff4– KorA, KorB dissociation rates, KorA for KorA-DNA complex, KorB from KorB-DNA complex, KorA from KorA-KorB-DNA complex and KorB from KorA-KorB-DNA complex, respectively, kA, kB – maximal KorA and KorB synthesis rates, kP – plasmid replication rate, πX, πY- scaling parameters for the protein synthesys, konD – protein association rate to the DNA, konP – protein dimerization rate. Smaller control coefficient implies greater robustness; model descriptions in figure 1b. These are mean control coefficients from 1000 re-samples of parameter values. Note that the values are very similar to those presented in Table 1, indicating that the results on robustness of the systems to changes in parameter values are themselves robust to uncertainty in the parameter values. (DOCX) [file pone.0049678.s003.docx]

|  | CCO | CCOnoC | CCOregB | CCOnoR |
| --- | --- | --- | --- | --- |
| *k_off_*_1_ | 0.005 | 0.004 | - | - |
| *k_off_*_2_ | 0.002 | 0.003 | 0.034 | - |
| *k_off_*_3_ | 0.065 | 0.058 | - | - |
| *k_off_*_4_ | 0.147 | 0.146 | - | - |
| *k*_A_ | 0.423 | 0.409 | 0.500 | 0.500 |
| *k*_B_ | -0.101 | -0.091 | -0.024 | 0.000 |
| *k*_P_ | 0.208 | 0.211 | 0.456 | 0.502 |
| π_X_ | 0.147 | 0.141 | - | - |
| π_Y_ | 0.065 | 0.077 | 0.410 | - |
| *k*_onD_ | -0.146 | -0.144 | -0.023 | - |
| *k*_onP_ | -0.001 | -0.002 | -0.001 | 0.001 |
