# Supplementary material for: Adaptation for Protein Synthesis Efficiency in a Naturally Occurring Self-Regulating Operon
Source: PLoS One. 2012 Nov 20;7(11):e49678. doi: 10.1371/journal.pone.0049678 (PMC3502259; doi:10.1371/journal.pone.0049678)
Supplement: Table S2 — KorA and KorB synthesis rates for different models. kA – KorA synthesis rate, kB - KorB synthesis rate; model descriptions in figure 1b. (DOCX) [file pone.0049678.s004.docx]

| Parameter | Value [ s^-1^] | Parameter | Value [ s^-1^] |
| --- | --- | --- | --- |
| k_A_ (CCO) | 11.500 | k_B_ (CCO) | 3.200 |
| k_A_ (CCOnoC) | 3.500 | k_B_ (CCOnoC) | 0.920 |
| k_A_ (CCOregB) | 0.205 | k_B_ (CCOregB) | 0.053 |
| k_A_ (CCOnoR) | 0.147 | k_B_ (CCOnoR) | 0.039 |
