# Supplementary material for: Adaptation for Protein Synthesis Efficiency in a Naturally Occurring Self-Regulating Operon
Source: PLoS One. 2012 Nov 20;7(11):e49678. doi: 10.1371/journal.pone.0049678 (PMC3502259; doi:10.1371/journal.pone.0049678)
Supplement: Table S3 — Parameter values for mRNA production analyses. kAi, kBi – KorA and KorB translation initiation rates respectively, kMi – transcription initiation rate, γMi– mRNA turn-over rate; model descriptions in figure 1b. (DOCX) [file pone.0049678.s005.docx]

| 'Transcription tuned' | | 'Translation tuned' | | |
| --- | --- | --- | --- | --- |
| Parameter | Value [s^-1^] | Parameter | Value [s^-1^] |  |
| k_Ai_ | 0.091000 | γ_Mi_ | 0.003000 |  |
| k_Bi_ | 0.025000 | k_Mi_ | 0.400000 |  |
| γ_Mi_ | 0.003000 | k_Ai_ (CCO) | 0.091000 |  |
| k_Mi_ (CCO) | 0.400000 | k_Bi_ (CCO) | 0.025000 |  |
| k_Mi_ (CCOnoC) | 0.135000 | k_Ai_ (CCOnoC) | 0.029800 |  |
| k_Mi_ (CCOregB) | 0.007700 | k_Bi_ (CCOnoC) | 0.008300 |  |
| k_Mi_ (CCOnoR) | 0.005550 | k_Ai_ (CCOregB) | 0.001740 |  |
|  |  | k_Bi_ (CCOregB) | 0.000482 |  |
|  |  | k_Ai_ (CCOnoR) | 0.001250 |  |
|  |  | k_Bi_ (CCOnoR) | 0.000345 |  |
