# Supplementary material for: Adaptation for Protein Synthesis Efficiency in a Naturally Occurring Self-Regulating Operon
Source: PLoS One. 2012 Nov 20;7(11):e49678. doi: 10.1371/journal.pone.0049678 (PMC3502259; doi:10.1371/journal.pone.0049678)
Supplement: Table S4 — Parameter values for analyses of regulatory mechanism evolution. kaff – an affinity of a transcription factor to the DNA strand, r – expression reduction, kAi, kBi – KorA and KorB synthesis rates respectively; model descriptions in Figure 1b. (DOCX) [file pone.0049678.s006.docx]

| k_aff_ [nM] | r | k_Ai_ [s^-1^] | k_Bi_ [s^-1^] |
| --- | --- | --- | --- |
| 3 | 0.00 | 0.08520 | 0.022000 |
| 3 | 0.25 | 0.00470 | 0.001200 |
| 3 | 0.50 | 0.00250 | 0.000600 |
| 3 | 0.75 | 0.00180 | 0.000450 |
| 3 | 1.00 | 0.00125 | 0.000345 |
| 10 | 0.00 | 0.02550 | 0.006000 |
| 10 | 0.25 | 0.00440 | 0.001000 |
| 10 | 0.50 | 0.00235 | 0.000560 |
| 10 | 0.75 | 0.00170 | 0.000400 |
| 10 | 1.00 | 0.00125 | 0.000345 |
| 30 | 0.00 | 0.00950 | 0.002200 |
| 30 | 0.25 | 0.00360 | 0.000900 |
| 30 | 0.50 | 0.00220 | 0.000500 |
| 30 | 0.75 | 0.00160 | 0.000400 |
| 30 | 1.00 | 0.00125 | 0.000345 |
| 100 | 0.00 | 0.00360 | 0.000800 |
| 100 | 0.25 | 0.00250 | 0.000600 |
| 100 | 0.50 | 0.00185 | 0.000400 |
| 100 | 0.75 | 0.00155 | 0.000350 |
| 100 | 1.00 | 0.00125 | 0.000345 |
| 300 | 0.00 | 0.00200 | 0.000400 |
| 300 | 0.25 | 0.00179 | 0.000390 |
| 300 | 0.50 | 0.00160 | 0.000350 |
| 300 | 0.75 | 0.00155 | 0.000350 |
| 300 | 1.00 | 0.00125 | 0.000345 |
